# Functional Differences Between SIRPα Splice Isoforms

**DOI:** 10.1111/gtc.70041

**Published:** 2025-08-05

**Authors:** Mihoko Kajita, Yojiro Matsui, Kotaro Sugimoto, Shuto Takeuchi, Shota Matsumoto, Takahiro Okumura, Hiroyuki Kajiura, Kazuki Motomura, Atsushi Takeda, Tomomi Koshiyama, Kyoko Shirakabe

**Affiliations:** ^1^ Department of Biomedical Sciences, College of Life Sciences Ritsumeikan University Kyoto Japan; ^2^ Department of Applied Chemistry, College of Life Sciences Ritsumeikan University Kyoto Japan; ^3^ International Center for Biotechnology Osaka University Osaka Japan; ^4^ Institute for Open and Transdisciplinary Research Initiatives (OTRI) Osaka University Osaka Japan; ^5^ Research Organization of Science and Technology Ritsumeikan University Kyoto Japan; ^6^ Department of Biotechnology, College of Life Sciences Ritsumeikan University Kyoto Japan; ^7^ Ritsumeikan Global Innovation Research Organization Ritsumeikan University Kyoto Japan

**Keywords:** “don't eat me” signal, CD47, macrophage, phagocytosis, SIRPα, splice isoform

## Abstract

Signal regulatory protein (SIRP) α, an inhibitory receptor belonging to the immunoglobulin (Ig) superfamily is abundantly expressed in phagocytes such as macrophages. CD47, the ligand for SIRPα, is expressed in most healthy cells, and called “don't eat me” signal because it binds to SIRPα on the surface of macrophages and inhibits phagocytosis. SIRPα has multiple splice isoforms, but most functional analyses have been carried out using long SIRPα, the SIRPα isoform with three extracellular Ig domains. In this study, we analyzed the expression and function of short SIRPα, an SIRPα isoform with only one extracellular Ig domain. In resting mouse macrophage Raw 264.7 cells, the short and long SIRPα mRNA expression levels were similar, and the proportion of short SIRPα mRNA decreased substantially after endotoxin stimulation. Short SIRPα bound to CD47 as same as long SIRPα, however, did not suppress the phagocytosis of recombinant CD47‐coated beads, unlike long SIRPα. These results suggest that short SIRPα may be a “don't eat me” signal regulator with different expression and function from long SIRPα.

## Graphical abstract

SIRPα is expressed in phagocytic cells, and when it recognizes CD47, “don't eat me” signal, it suppresses phagocytosis. We show here that short SIRPα, a less‐studied splice isoform, has a different function from a well‐studied long SIRPα. Short SIRPα may act as a “don't eat me” signal regulator.

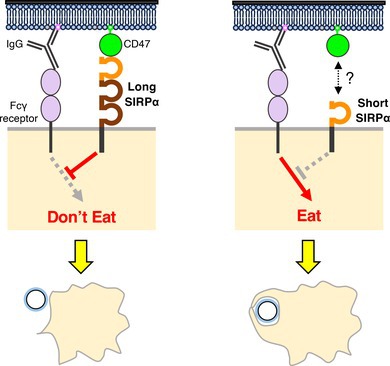

## 1 Introduction

To maintain homeostasis within the body, phagocytosis must be strictly regulated so that healthy cells are not unnecessarily eliminated (Arandjelovic and Ravichandran 2015). Signal regulatory protein (SIRP) α is an inhibitory receptor belonging to the immunoglobulin (Ig) superfamily that is expressed abundantly in phagocytes including macrophages and negatively regulates phagocytosis (Barclay and Brown 2006; Matozaki et al. 2009). The ligand for SIRPα, CD47, is called “don't eat me” signal, is expressed on most healthy cells, and prevents the healthy cells from being phagocytosed by binding to SIRPα on the surface of macrophages (Matozaki et al. 2009; Barclay and Van den Berg 2014; Kelley and Ravichandran 2021). The importance of the CD47–SIRPα signaling was first observed in the phagocytosis of mature red blood cells by spleen macrophages. In wild‐type mice, CD47‐deficient red blood cells were phagocytosed more rapidly than wild‐type ones (Oldenborg et al. 2000). Subsequent research has shown that CD47–SIRPα signaling can inhibit phagocytosis activated by various factors such as IgG, complement factors, phosphatidylserine, and calreticulin (Oldenborg et al. 2001; Gardai et al. 2005; Morrissey et al. 2020). Since elevated CD47 mRNA expression correlates with poor prognosis of human cancers (Majeti et al. 2009; Willingham et al. 2012), CD47–SIRPα signaling plays an important role in cancer immune evasion and is a target for cancer immunotherapy (Chao et al. 2012; Alvey and Discher 2017; Veillette and Chen 2018).

Several groups, including ours, have reported that SIRPα is a target of ectodomain shedding (shedding), a processing mechanism for transmembrane proteins in which the juxtamembrane region is cleaved and almost the entire extracellular domain is solubilized (Ohnishi et al. 2004; Toth et al. 2013; Londino et al. 2015; Shirakabe et al. 2017). SIRPα shedding is activated by pro‐inflammatory stimuli such as bacterial endotoxin lipopolysaccharide (LPS), and contributes to the induction of inflammatory responses through various molecular mechanisms (Londino et al. 2015). We have previously shown that alternative splicing generates both shedding‐susceptible and shedding‐resistant SIRPα isoforms. The isoform having three extracellular Ig domains, hereafter referred to as long SIRPα, is shedding‐susceptible, while the isoform having only one Ig domain, short SIRPα, is shedding‐resistant (Shirakabe et al. 2017). Since most functional analyses of SIRPα have been carried out using long SIRPα, the function of short SIRPα has hardly been elucidated although its expression has been observed in various tissues and cells (Comu et al. 1997; Veillette et al. 1998; Sano et al. 1999; Shirakabe et al. 2017). In this study, we report our analysis on short SIRPα expression and function, which suggests that short SIRPα may regulate the “don't eat me” signal.

## 2 Results

### 2.1 SIRPα Isoform mRNA Expression Ratio Changes in Response to Endotoxin Stimulation

**FIGURE 1:**
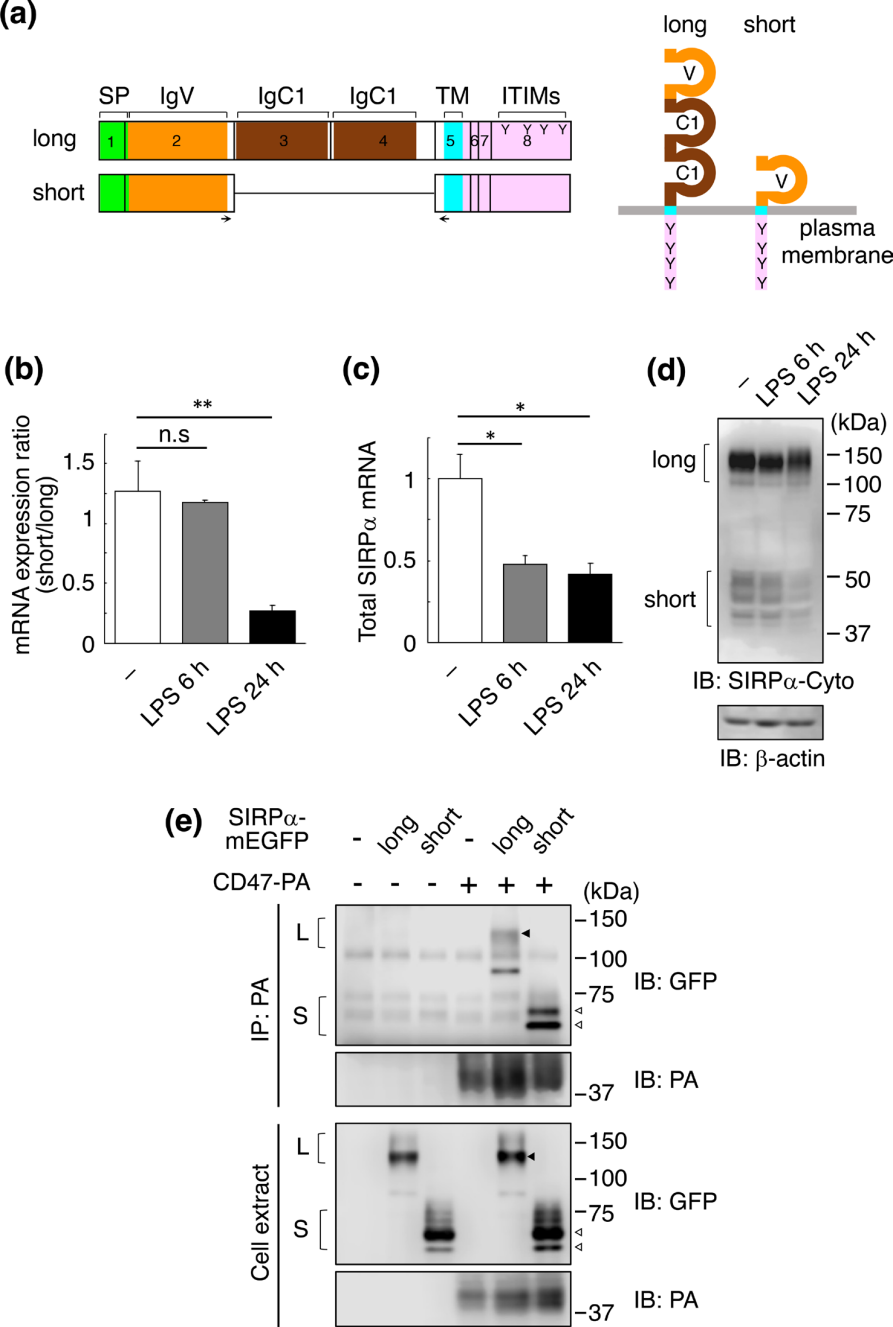
mRNA expression ratio and CD47 binding ability of signal regulatory protein (SIRP) α isoforms. (a) Schematic diagrams of splice isoforms of SIRPα. Exons are indicated by black rectangles with numbers (left). IgC1, Ig‐like constant 1‐type domain (brown); IgV, immunoglobulin (Ig)‐like variable‐type domain (orange); ITIMs, immunoreceptor tyrosine‐based inhibitory motifs; SP, signal peptide (green); TM, transmembrane domain (light blue). Y characters indicate tyrosine residues in ITIMs. Cytoplasmic domains are colored in pink. Arrows indicate the approximate positions of the primers used in RT‐PCR. Topology of SIRPα isoforms is shown on the right. (b) mRNA expression ratio of SIRPα isoforms in LPS‐treated Raw 264.7 cells. Raw 264.7 cells were treated with 100 ng/mL LPS for the indicated time, and RT‐PCR was performed using the collected RNA to calculate the mRNA expression ratio of SIRPα isoforms. Mean values from three independent experiments are shown with SE. Asterisks indicate the statistical significance (*p* < 0.01) as determined by one‐way ANOVA followed by Dunnett's test. n.s. indicates not significant. (c) Total amount of SIRPα mRNA in LPS‐treated Raw 264.7 cells. Raw 264.7 cells were treated with LPS for the indicated time, and quantitative RT‐PCR was performed using primers common to both SIRPα isoforms. Mean values from three independent experiments are shown with SEM. Asterisks indicate the statistical significance (*p* < 0.05) by one‐way ANOVA with Dunnett's test. (d) Levels of SIRPα isoform proteins in LPS‐treated Raw 264.7 cells. Cell extracts were subjected to western blotting using an anti‐SIRPα antibody that recognizes the cytoplasmic domain of SIRPα (SIRPα‐Cyto). Loading control: β‐actin. (e) CD47 binding ability of SIRPα isoforms. Chinese hamster ovary (CHO) cells were co‐transfected with expression vectors for C‐terminally monomeric EGFP (mEGFP)‐fused long or short SIRPα—and CD47–PA as indicated. Interaction was detected by immunoprecipitation (IP) with anti‐PA antibody, followed by western blotting (IB) with anti‐GFP or anti‐PA antibody. Total cell extracts were also immunoblotted with indicated antibodies. Black and white triangles indicate the bands corresponding to long SIRPα (L) and short SIRPα (S), respectively.

We have already shown that both long and short SIRPα protein expression levels are comparable in Raw 264.7 cells (Shirakabe et al. 2017). In this study, we investigated the mRNA expression ratio of these isoforms using RT‐PCR. Long SIRPα is encoded by eight exons, while short SIRPα lacks exons 3 and 4, which encode the two extracellular IgC1 domains (Figure 1a). Therefore, we performed RT‐PCR using primers specific for SIRPα exons 2 and 5 (Figure 1a, arrows), and calculated the mRNA expression ratio by separating and quantifying each SIRPα isoform cDNA fragment using electrophoresis. In unstimulated Raw 264.7 cells, the mRNA expression for the two SIRPα isoforms was similar, but the proportion of short SIRPα mRNA decreased significantly with LPS stimulation in proportion to the stimulation time (Figure 1b). To confirm changes in the total amount of SIRPα mRNA, qPCR was performed using primers specific for exons 6, 7, and 8, which are common to both SIRPα isoforms. The results showed that the total amount of SIRPα mRNA decreased by half within 6 h after LPS stimulation and remained at this level until 24 h (Figure 1c). Furthermore, to investigate the expression of SIRPα isoform proteins, western blotting was performed using an antibody that recognizes both SIRPα isoforms. The results showed that the expression levels of both SIRPα isoforms decreased at 6 h after LPS stimulation, and the expression level of short SIRPα further decreased at 24 h (Figure 1d). These results suggest that LPS stimulation reduces the expression of both long and short SIRPα, but the reduction of short SIRPα expression is more pronounced than that of long SIRPα.

### 2.2 CD47‐Binding Ability of SIRPα Isoforms

Next, we investigated the binding ability of SIRPα isoforms to CD47, the ligand for SIRPα. C‐terminally monomeric EGFP (mEGFP)‐fused SIRPα isoforms were expressed in Chinese hamster ovary (CHO) cells with or without C‐terminally PA‐tagged CD47, and the binding of SIRPα isoforms to CD47 was examined by co‐immunoprecipitating SIRPα isoforms using an anti‐PA antibody. Western blotting showed that both long and short SIRPα–mEGFP were co‐precipitated with CD47–PA (Figure 1e). These results suggest that the extracellular two IgC1 domains of long SIRPα are not required for binding to CD47, which is consistent with the results of previous reports indicating that the N‐terminal IgV domain of long SIRPα is responsible for the binding to CD47 (Vernon‐Wilson et al. 2000; Seiffert et al. 2001; Liu et al. 2004).

### 2.3 Generation of Macrophage Cells Expressing Long or Short SIRPα–mEGFP Instead of Endogenous SIRPα

**FIGURE 2:**
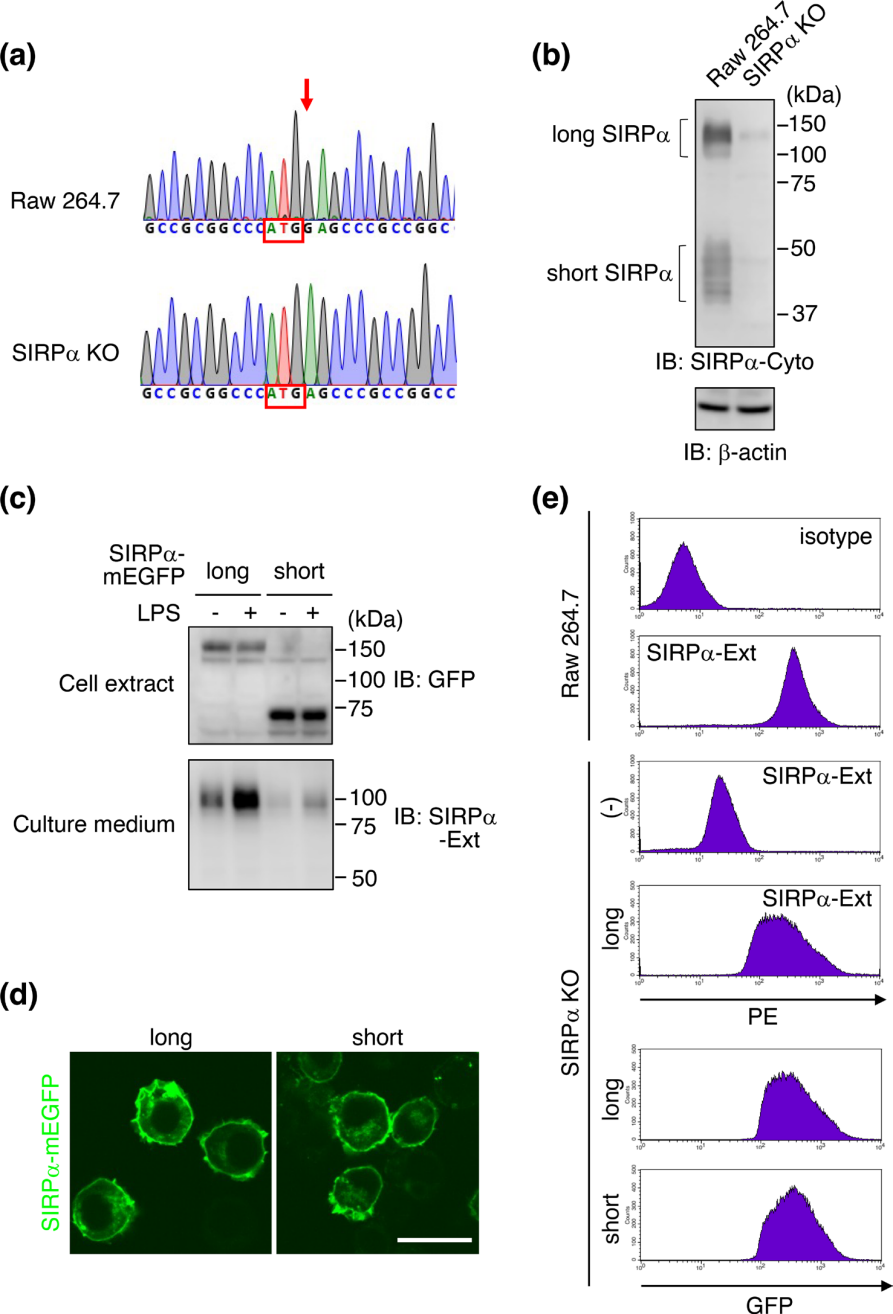
Generation of macrophage cells expressing C‐terminally monomeric EGFP (mEGFP) fused signal regulatory protein (SIRP) α isoforms instead of endogenous SIRPα. (a) Genomic sequence of Raw 264.7 and SIRPα KO cells. Red arrow indicates the base deleted in SIRPα KO cells. Start codon is marked with red rectangles. (b) Western blotting of cell extracts from Raw 264.7 and SIRPα KO cells using an anti‐SIRPα antibody that recognizes the cytoplasmic domain of SIRPα (SIRPα‐Cyto). Loading control: β‐actin. (c) SIRPα KO cells expressing long or short SIRPα–mEGFP were treated with (+) or without (−) 1 μg/mL of LPS for 60 min. Both cell extracts (cell extract) and culture supernatants (culture medium) were subjected to western blotting using indicated antibodies. SIRPα‐Ext indicates the antibody that recognizes the extracellular domain of SIRPα. (d) Cell surface localization of long and short SIRPα–mEGFP. SIRPα KO cells were infected with a lentiviral vector expressing long or short SIRPα–mEGFP and observed using a confocal microscope. Scale bar: 20 μm. (e) Flow cytometry analysis of Raw 264.7 and SIRPα KO cells expressing long or short SIRPα–mEGFP using a PE‐labeled antibody that recognizes the extracellular domain of SIRPα (SIRPα‐Ext) or fluorescence signal of mEGFP (bottom two panels).

To analyze SIRPα isoform localization and function, we generated macrophage cells lacking endogenous SIRPα and expressing each SIRPα isoform as an mEGFP fusion protein using Raw 264.7 cells as the material. First, we introduced mutations near the start codon of the *Sirpa* gene in Raw 264.7 cells using the CRISPR/Cas9 system and cloned the mutated cells. When the genomic DNA sequences of the obtained clones were examined, one clone had a deletion of one nucleotide immediately after the start codon in both alleles (Figure 2a, red arrow). Since this deletion causes the expression of a 10‐amino acid peptide that is unrelated to SIRPα, we examined the expression of SIRPα protein by western blotting and found that this mutant clone expressed almost no endogenous SIRPα (Figure 2b). We hereafter refer to the clone as SIRPα KO cells.

Next, we constructed lentiviral vectors expressing long or short SIRPα–mEGFP in SIRPα KO cells. To avoid cleavage by CRISPR/Cas9 in SIRPα KO cells, we first constructed expression vectors encoding mEGFP‐fused SIRPα isoforms by replacing the endogenous signal sequence of SIRPα, which is susceptible to CRISPR/Cas9, with an exogenous one. Western blotting using the extracts of SIRPα KO cells transfected with the constructed vectors confirmed both long and short SIRPα–mEGFP expression (Figure 2c, upper panel). Due to differences in glycosylation, SIRPα–mEGFP expressed in Raw 264.7 cells exhibited lower electrophoretic mobility on SDS‐PAGE compared to the same protein expressed in CHO cells (Figure 1e and 2c). In addition, western blotting using the culture medium showed that the soluble extracellular domain was released from long SIRPα–mEGFP‐expressing cells in response to LPS stimulation, suggesting that the shedding susceptibility of long SIRPα is maintained after fusion with mEGFP (Figure 2c, lower panel). Due to the presence of residual endogenous SIRPα in SIRPα KO cells, a small amount of the soluble extracellular domain was detected in the culture medium of short SIRPα–mEGFP‐expressing cells (Figure 2c, lower panel). The cDNAs of these expression vectors were amplified and inserted into a lentiviral vector plasmid to produce lentiviral vectors, which were then infected into SIRPα KO cells. Confocal microscopy revealed that both isoforms were predominantly expressed at the cell membrane, with weaker expression also observed within the cell (Figure 2d). Flow cytometry analysis using an antibody recognizing the extracellular domain of SIRPα (SIRPα‐Ext) showed that the amount of long SIRPα–mEGFP expressed on the SIRPα KO cell surface was almost equivalent to that of endogenous SIRPα in Raw 264.7 cells (Figure 2e, second and fourth panels). Since this antibody does not recognize short SIRPα, short and long SIRPα–mEGFP expression was compared using the mEGFP‐derived fluorescence signal. The fluorescence signal of the cells expressing short and long SIRPα–mEGFP was similar (Figure 2e, bottom two panels), indicating that both long and short SIRPα–mEGFP expression level in SIRPα KO cells was similar to the endogenous SIRPα expression level in Raw 264.7 cells. When directly compared, the median fluorescence intensity of cells expressing short SIRPα–mEGFP was higher than that of cells expressing long SIRPα–mEGFP (Figure 2e, bottom two panels). This observation is consistent with the western blotting results, which showed higher expression levels of short SIRPα–mEGFP compared to long SIRPα–mEGFP (Figure 2c).

### 2.4 Preparation of Phagocytosis Target Beads Coated With CD47 and an “Eat Me” Signal, IgG

**FIGURE 3:**
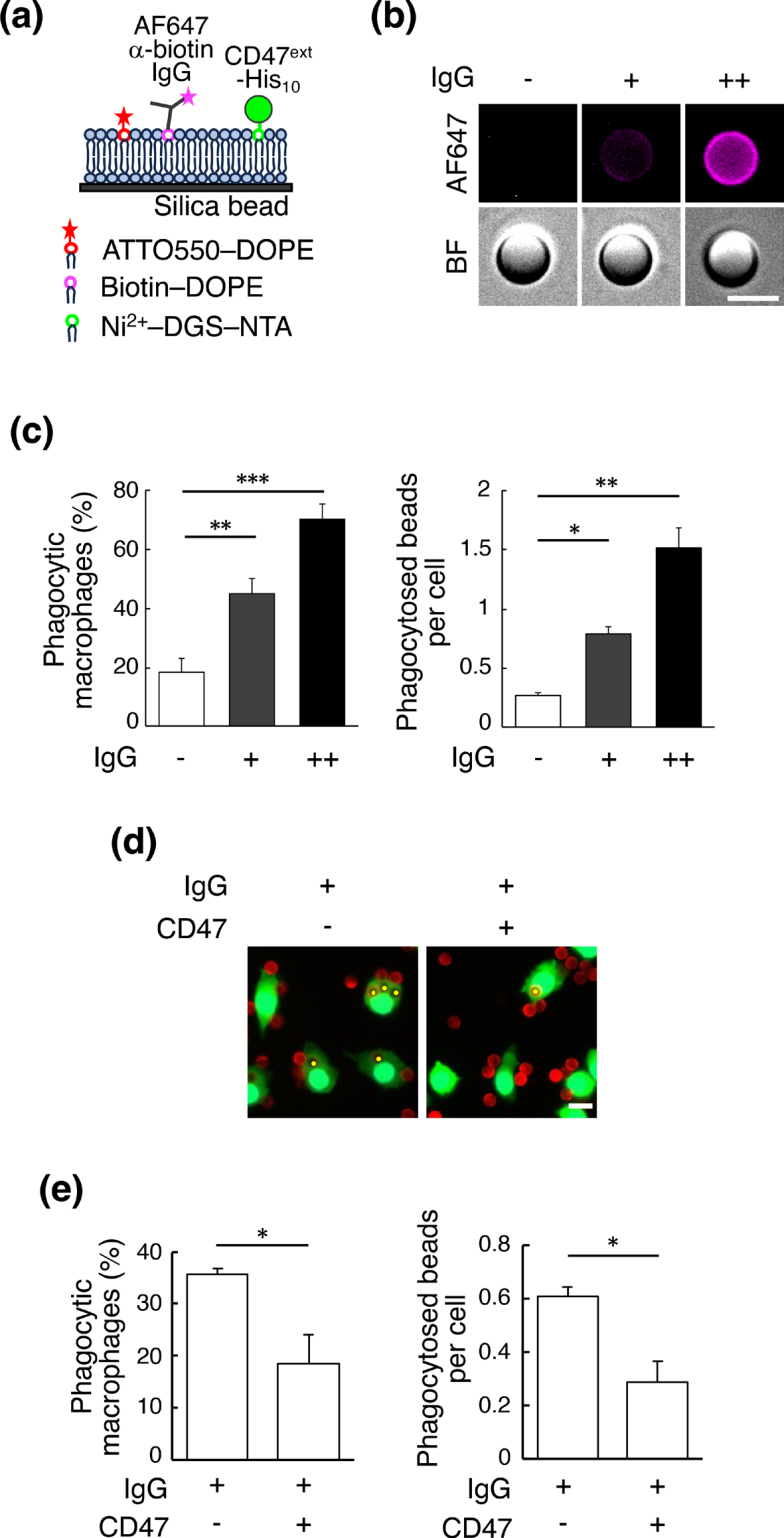
Phagocytic target bead conjugated with IgG and CD47 via a supported lipid bilayer. (a) Schematic diagram of a phagocytic target bead surface used in this study. The beads are covered with a supported lipid bilayer, and IgG and CD47 were bound to the constituent lipid heads. Alexa Fluor 647–anti‐biotin IgG is bound to biotin–DOPE, and His_10_‐tagged extracellular domain of CD47 is bound to Ni^2+^–DGS–NTA. (b) Fluorescence images of target beads conjugated with IgG. 300 (+) or 3000 (++) Alexa Fluor 647–anti‐biotin IgG molecules/μm^2^ were conjugated to the beads. AF647 indicates the signal of Alexa Fluor 647, and BF indicates bright field images. Scale bar: 5 μm. (c) The percentage of Raw 264.7 cells that phagocytosed IgG‐coated beads (left) and the number of phagocytosed beads divided by the number of counted cells (right). Beads conjugated with IgG at the indicated density (300 (+) or 3000 (++) molecules/μm^2^) and beads without conjugation (−) were added to EGFP‐expressing Raw 264.7 cells; the cells were fixed after 30 min and observed under a fluorescence microscope to count the phagocytic cells and phagocytosed beads. Mean values from three independent experiments are shown with SEM. Asterisks indicate the statistical significance (*p < 0.05, **p < 0.01, ***p < 0.001) as determined by one‐way ANOVA with Dunnett's test. (d) Phagocytosis of IgG‐ or IgG/CD47‐coated beads by Raw 264.7 cells. IgG (300 molecules/μm^2^) and/or CD47 (6000 molecules/μm^2^)‐conjugated beads were added to EGFP‐expressing Raw 264.7 cells, which were fixed after 30 min and observed under a fluorescence microscope. The supported lipid bilayers contain ATTO550–DOPE (red), Raw 264.7 cells express EGFP (green), and the nuclei are stained with Hoechst 33342 (cyan). Internalized beads are indicated with a yellow dot. Scale bar: 10 μm. (e) The percentage of Raw 264.7 cells that phagocytosed IgG‐ or IgG/CD47‐coated beads (left) and the number of phagocytosed beads divided by the number of counted cells (right). Mean values from three independent experiments are shown with SEM. Asterisks indicate the statistical significance (*p* < 0.05) as calculated by Student's *t*‐test.

We then prepared phagocytosis target beads coated with CD47 and an “eat me” signal, IgG, since CD47 can inhibit phagocytosis activated by IgG (Morrissey et al. 2020). To allow CD47 and IgG to move around on the bead surface, the silica beads were coated with a supported lipid bilayer, and CD47 and IgG were bound to the head of the constituent phospholipids, as reported previously (Morrissey et al. 2020) (Figure 3a). Coated beads were added to Raw 264.7 cells expressing EGFP, and after 30 min the cells were fixed and observed under a fluorescence microscope to calculate the “percentage of cells that phagocytosed the beads” and the “number of phagocytosed beads divided by the number of counted cells”. First, we confirmed that increasing the amount of IgG on the bead surface enhanced bead phagocytosis by Raw 264.7 cells (Figure 3b,c). We then confirmed that phagocytosis activated by a moderate amount of IgG was inhibited by CD47 (Figure 3d,e). The phagocytosis inhibition by CD47 was not observed in SIRPα KO cells (data not shown). These results show that CD47 on the prepared bead surface can inhibit phagocytosis through binding to SIRPα.

### 2.5 SIRPα Isoform Localization in Phagocytic Cups

**FIGURE 4:**
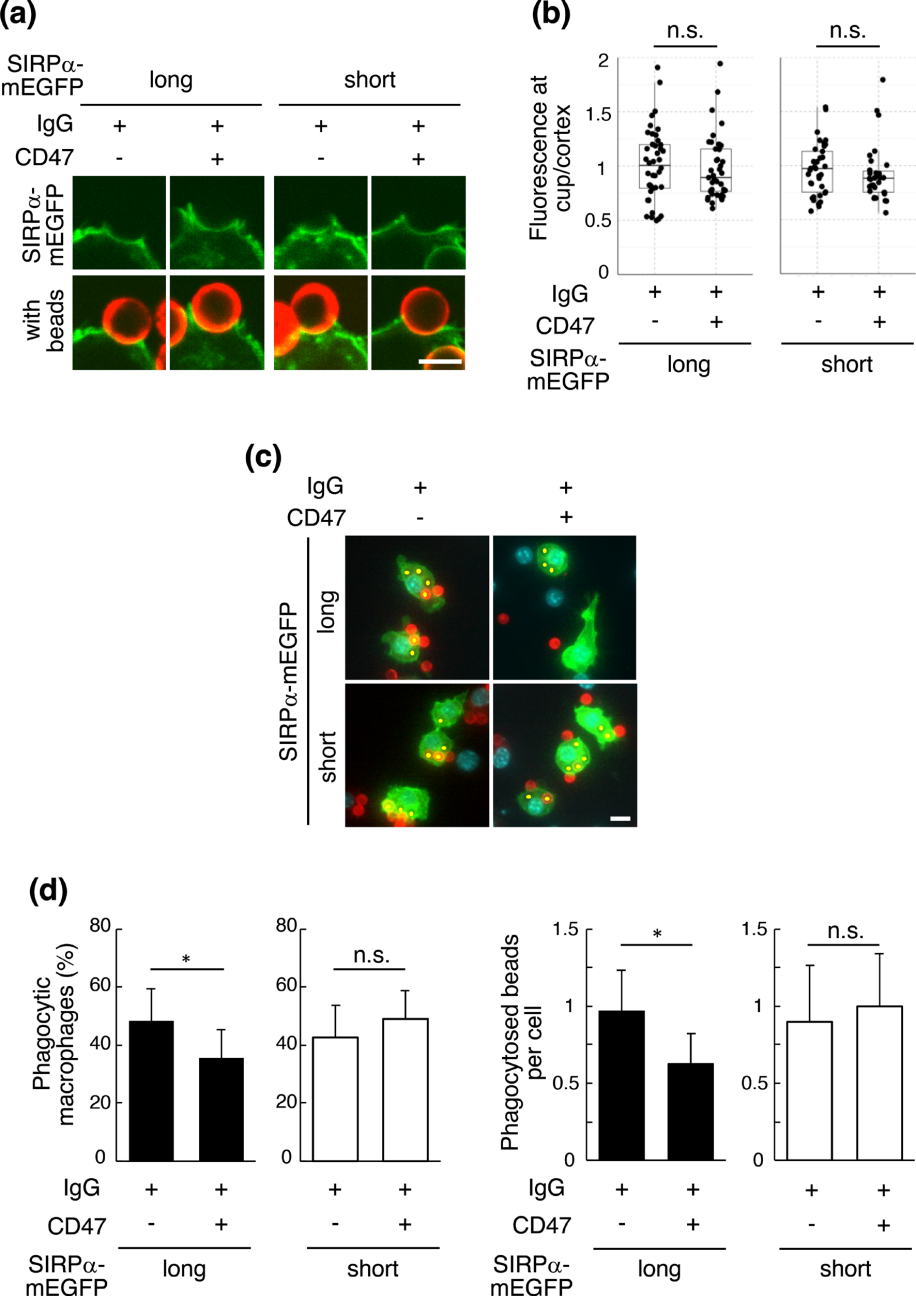
Short signal regulatory protein (SIRP) α does not inhibit phagocytosis, unlike long SIRPα. (a) SIRPα isoform localization in phagocytic cups. IgG (300 molecules/μm^2^) and/or CD47 (6000 molecules/μm^2^)‐conjugated beads were added to long or short SIRPα–C‐terminally monomeric EGFP (mEGFP)‐expressing cells, and cells were fixed after 15 min and observed under a confocal microscope. The supported lipid bilayers contain ATTO550–DOPE (red), and the macrophages express long or short SIRPα–mEGFP (green). Scale bar: 5 μm. (b) The ratio of mEGFP fluorescence at the phagocytic cup compared to that at the cortex for long and short SIRPα–mEGFP. Each dot represents an individual phagocytic cup. The data was collected from three independent experiments. n.s. indicates not significant. (c) Phagocytosis of IgG‐ or IgG/CD47‐coated beads by long or short SIRPα–mEGFP‐expressing cells. IgG‐ or IgG/CD47‐coated beads were added to long or short SIRPα–mEGFP‐expressing cells, and cells were fixed after 30 min and observed under a fluorescence microscope. The supported lipid bilayers contain ATTO550–DOPE (red), the macrophages express long or short SIRPα–mEGFP (green), and the nuclei are stained with Hoechst 33342 (cyan). Internalized beads are indicated with a yellow dot. Scale bar: 10 μm. (d) The percentage of macrophages that phagocytosed IgG‐ or IgG/CD47‐coated beads (left) and the number of phagocytosed beads divided by the number of counted cells (right). Mean values from three independent experiments are shown with SEM. Asterisks indicate the statistical significance (*p* < 0.05) as calculated by Student's *t*‐test. n.s. indicates not significant.

Morrissey et al. reported that long SIRPα, which has a large extracellular domain, tends to be excluded from the phagocytic cup where two membranes are closely attached, but when CD47 is present on the surface of targets, long SIRPα binds to CD47 and remains within the phagocytic cup, inhibiting phagocytosis (Morrissey et al. 2020). Since their report suggests that the localization in the phagocytic cup is important for SIRPα to inhibit phagocytosis, we investigated the localization of SIRPα isoforms in the phagocytic cup using the prepared cells and beads. We added IgG‐ or IgG/CD47‐coated beads to long or short SIRPα–mEGFP‐expressing cells, and the cells were fixed after 15 min and observed under a confocal microscope to quantify SIRPα isoform localization. In contrast to Morrissey's study, the localization of long SIRPα–mEGFP in phagocytic cups did not increase in the presence of CD47, which was the same as that of short SIRPα–mEGFP (Figure 4a,b). On the contrary, the localization of both SIRPα isoforms in the phagocytic cup tended to weaken in the presence of CD47.

### 2.6 SIRPα Isoform Activity to Inhibit Phagocytosis

We then examined whether SIRPα isoforms inhibited the phagocytosis of CD47‐conjugated beads. IgG‐ or IgG/CD47‐coated beads were added to long or short SIRPα–mEGFP‐expressing cells, the cells were fixed after 30 min and observed under a fluorescence microscope to quantify phagocytosis. Phagocytosis of beads by long SIRPα–mEGFP‐expressing macrophages was suppressed in the presence of CD47 (Figure 4c, top panels and 4d, black bars), indicating that long SIRPα responds to CD47 and inhibits phagocytosis. In contrast, phagocytosis of beads by short SIRPα–mEGFP‐expressing macrophages was not suppressed in the presence of CD47 (Figure 4c, bottom panels and 4d, white bars), suggesting that short SIRPα may not respond to CD47 and does not inhibit phagocytosis.

## 3 Discussion

In this study, we provided new insights into the expression and function of short SIRPα, an isoform that has not been well studied. Short SIRPα has CD47‐binding ability like long SIRPα, but the mRNA expression and the phagocytosis inhibitory activity are different. These findings suggest that while long SIRPα acts as a signal transducer, short SIRPα may act as a “don't eat me” signal regulator. Short SIRPα is also expressed in mouse peritoneal and bone marrow‐derived macrophages (data not shown), suggesting that it may contribute to the regulation of the “don't eat me” signal in various phagocytic cells. CD47–SIRPα signaling is involved in the development of diseases including cancer, and since short SIRPα is expressed as widely as long SIRPα, we believe that these findings will provide important perspectives into the mechanisms of onset and the development of treatments for these diseases. Specifically, diagnostic methods that estimate the phagocytic ability of disease‐associated phagocytes by analyzing the ratio of SIRPα isoforms, and therapeutic methods that regulate phagocytic ability by manipulating the ratio of SIRPα isoforms in phagocytes may be established. Elucidation of the molecular mechanism that determines the ratio of SIRPα isoforms will be an important challenge.

Short SIRPα binds to CD47 in the same manner as long SIRPα, and its cytoplasmic domain is identical to that of long SIRPα, suggesting that short SIRPα may also bind to CD47 and send a “don't eat me” signal. Why short SIRPα–mEGFP did not inhibit the phagocytosis of CD47‐coated beads, unlike long SIRPα–mEGFP, is currently unknown, but there are several possibilities. One possibility is the “physical size” of the extracellular domain. Since all beads used in this study were coated with IgG, the bead membrane and macrophage membrane should be at least approximately 12 nm apart based on the reported size of the Fcγ receptor (FcγR)–IgG complex (Bakalar et al. 2018; Suter et al. 2021). Although FcγR and SIRPα are in close proximity within the phagocytic cup (Tsai and Discher 2008; Morrissey et al. 2020), the length of the long SIRPα and CD47 complex is 13–14 nm (Hatherley et al. 2009; Suter et al. 2021), so the long SIRPα should bind sufficiently to CD47 on the beads in our system. On the contrary, the extracellular domain of short SIRPα is only one third that of long SIRPα, and may be too small to bind to CD47 on the bead surface. Binding to CD47 induces tyrosine phosphorylation of the intracellular immunoreceptor tyrosine‐based inhibition motif (ITIM) of long SIRPα, and recruit tyrosine phosphatases SHP‐1 and SHP‐2 (Fujioka et al. 1996; Kharitonenkov et al. 1997; Veillette et al. 1998; Okazawa et al. 2005). We have obtained preliminary results indicating that inhibition of tyrosine phosphatase enhances tyrosine phosphorylation of short SIRPα, suggesting that ITIM of short SIRPα is a target for tyrosine phosphorylation (data not shown). In the future, it will be possible to verify this possibility by adding CD47‐ or CD47/IgG‐coated beads to short SIRPα–mEGFP‐expressing cells and clarifying the differences in ITIM phosphorylation or tyrosine phosphatase recruitment.

Another possibility is the “*cis*‐interaction” with CD47. CD47 is also expressed on macrophages, and SIRPα on the macrophages binds to CD47 of the same cell; this *cis*‐interaction consistently suppresses the phagocytic activity of resting macrophages (Hayes et al. 2020). Considering the transfection efficiency, it is likely that our co‐precipitation experiments are detecting *cis*‐interactions of SIRPα and CD47. In addition, the co‐precipitation efficiency of short SIRPα is slightly higher than that of long SIRPα. Short SIRPα may not have been able to interact with CD47 on the beads because it strongly interacts with CD47 of the same cell in *cis*.

The other possibility is the “homodimerization.” The two IgC1 domains of long SIRPα contribute to its homodimer formation (Lee et al. 2010), so short SIRPα, which lacks the two IgC1 domains, may not form a homodimer. If SIRPα needs to dimerize to inhibit phagocytosis, the inability to form a homodimer could explain why short SIRPα cannot inhibit phagocytosis.

The last possibility we should mention is the “shedding susceptibility.” The soluble extracellular domain of SIRPα released by shedding inhibits the phagocytic activity of macrophages (Shen et al. 2022). Shedding‐resistant short SIRPα may be less effective at inhibiting phagocytosis than long SIRPα, because it does not release its extracellular domain. Investigating these possibilities will be a topic for future research.

Unlike the previous report (Morrissey et al. 2020), we did not observe long SIRPα retention in the phagocytic cup of CD47‐coated beads. The biggest difference between their experiment and ours is the presence of endogenous SIRPα in macrophages. We deleted endogenous SIRPα to accurately detect the exogenous SIRPα isoform localization, but they did not. In addition, there are differences in the substrate on which the cells were seeded. We used fibronectin coating to facilitate observation of cell contours, but Morrissey et al. did not appear to use it. Fibronectin has been reported to promote tyrosine phosphorylation of SIRPα (Fujioka et al. 1996). Basal activation of SIRPα may have weakened its binding to CD47 and inhibited its retention in the phagocytic cup in our system. In any case, our findings raise the possibility that the localization of SIRPα may not be directly correlated with the transmission of the “don't eat me” signal. To determine which observation is closer to the physiological state, the localization of endogenous SIRPα, and if possible, of both isoforms, should be identified in the phagocytic cups.

The amount of mRNA of long and short SIRPα was similar in resting Raw 264.7 cells, but the proportion of short SIRPα mRNA significantly decreased in response to LPS stimulation. These results suggest that alternative splicing of SIRPα transcripts can change in response to external conditions. As mentioned above, if short SIRPα has a stronger *cis*‐interaction than long SIRPα and strongly suppresses phagocytosis, then macrophages may need to suppress its expression and activate phagocytosis during inflammatory responses. The changes in alternative splicing are involved in various diseases, including cancer (Baralle and Giudice 2017; Bradley and Anczuków 2023). Investigating whether alternative SIRPα splicing and phagocytic activity of macrophages are altered in these diseases might help to elucidate their pathogenesis.

## 4 Experimental Procedures

### 4.1 Cell Line, Transfection, and Sample Preparation for Western Blotting

RAW 264.7 cells were cultured in high‐glucose DMEM supplemented with 10% fetal bovine serum (FBS), 50 μM 2‐mercaptoethanol, 100 units/mL penicillin G, and 100 μg/mL streptomycin. Human embryonic kidney (HEK) 293 T cells were cultured in high‐glucose DMEM supplemented with 10% FBS, 100 units/mL penicillin G, and 100 μg/mL streptomycin. Chinese hamster ovary (CHO) cells were cultured in alpha‐MEM supplemented with 10% FBS, 100 units/mL penicillin G, and 100 μg/mL streptomycin. Sf9 cells were cultured in Sf‐900 III SFM (Thermo Fisher Scientific, Waltham, MA, USA) supplemented with 10% FBS, 100 units/mL penicillin G, and 100 μg/mL streptomycin. Transfection was performed using FuGENE HD (Promega, Madison, WI, USA) for RAW 264.7 and HEK293T cells and PEI‐MAX (Polysciences, Warrington, PA, USA) for CHO cells. For western blotting, cell extracts and proteins from the culture supernatants were prepared as described previously (Shirakabe et al. 2014). For immunoprecipitation, the cells were lysed using an immunoprecipitation buffer [20 mM Tris–HCl (pH 7.5), 150 mM NaCl, 2 mM EDTA, 1% Nonidet P‐40] supplemented with a protease inhibitor cocktail (Sigma‐Aldrich, St. Louis, MO, USA) and 10 μM BB94 (Selleck Chemicals, Houston, TX, USA) and PA‐tagged proteins were immunoprecipitated using anti‐PA tag antibody beads (Fujifilm Wako, Tokyo, Japan).

### 4.2 Antibodies, Chemicals, Expression Vectors and Lentiviral Vectors

Antibodies were purchased from BioLegend (anti‐SIRPα (Ext), 144001; PE‐anti‐SIRPα, 144011; PE Rat IgG1, 400,407; San Diego, CA, USA), ProSci (anti‐SIRPα (Cyto), 1125; Poway, CA, USA), Takara Bio (anti‐GFP, 632380; Shiga, Japan), Fujifilm Wako (anti‐PA tag, NZ‐1), Santa Cruz Biotechnology (anti‐β‐actin, sc‐81,178; Dallas, TX, USA), BD Biosciences (anti‐CD16/CD32, 553141; Franklin Lakes, NJ, USA), and Jackson ImmunoResearch (Alexa Fluor 647–anti‐biotin IgG, 200–602‐211; West Grove, PA, USA). LPS was purchased from Sigma‐Aldrich. 7‐aminoactinomycin D was purchased from BD Biosciences. 16:0–18:1 PC (POPC), 18:1 PEG5000 PE, 18:1 biotinyl Cap PE, and 18:1 Ni^2+^–DGS–NTA were purchased from Avanti Research (Alabaster, AL, USA). ATTO 550–DOPE was purchased from ATTO‐TEC GmbH (Siegen, Germany). Hoechst 33342 was purchased from Thermo Fisher Scientific. The SIRPα isoform coding sequences were subcloned into pmEGFP‐N1 expression vector containing the signal sequence of IL‐6, after removal of their signal sequences (Met^1^‐Gly^31^). The cDNA for each mEGFP‐fused SIRPα isoform was amplified by PCR and subcloned into pLenti CMV GFP Puro (Addgene, Watertown, MA, USA) using In‐Fusion technology (Takara Bio).

### 4.3 Quantification of mRNA of SIRPα Isoforms

Total RNA was extracted from Raw 264.7 cells treated with 100 ng/mL LPS for 6–24 h, and SIRPα cDNA fragments corresponding to exons 2–5 were amplified using RT‐PCR with primers 5′‐ATACAATCTGGAGGGGGAACAGAG‐3′ and 5′‐CCGATGAAGACATTCCAGTTGTGG‐3′. The PCR products were separated and quantified using a bioanalyzer 2100 (Agilent, Santa Clara, CA, USA). Quantitative RT‐PCR was performed using PowerUp SYBR Green Master Mix (Thermo Fisher Scientific) on a StepOne Real‐Time PCR system (Thermo Fisher Scientific). Relative gene expression levels were calculated using the ddCt method with GAPDH as the endogenous normalization control. Primer sequences were as follows: SIRPα, sense, 5′‐ACATCTTCCACACGGTTGCA‐3′, antisense, 5′‐GCTTCTTCTCTTTGGGCAGA‐3′, GAPDH, sense, 5′‐AATGTGTCCGTCGTGGATCT‐3′, antisense, 5′‐CATCGAAGGTGGAAGAGTGG‐3′.

### 4.4 Generation of Macrophage Cells Expressing mEGFP‐Fused SIRPα Isoforms

A lentiviral vector for CRISPR/Cas9‐based genome editing was prepared as reported previously (Iwagishi et al. 2020). The oligonucleotide sequences used in this study were 5′‐CACCGGCCGGGCCGGCGGGCTCCAT‐3′ and 5′‐AAACATGGAGCCCGCCGGCCCGGCC‐3′. Raw 264.7 cells infected with the CRISPR/Cas9 lentiviral vector were cloned, and the genome of multiple clones was sequenced. A clone with a deletion of one base immediately after the start codon of the *Sirpa* gene in both alleles was defined as SIRPα KO cells. SIRPα KO cells were further infected with lentiviral vectors expressing long or short SIRPα–mEGFP and used within a week after infection. Flow cytometry of long or short SIRPα–mEGFP‐expressing cells was performed using BD FACSCalibur (BD Biosciences).

### 4.5 Supported Lipid Bilayer‐Coated Silica Bead Preparation

The supported lipid bilayer‐coated silica beads were prepared using a method modified from those described in previous reports (Bakalar et al. 2018; Joffe et al. 2020; Morrissey et al. 2020). A chloroform solution containing 96.8% POPC, 0.1% 18:1 PEG5000 PE, 1% 18:1 biotinyl Cap PE, 2% 18:1 Ni^2+^–DGS–NTA, and 0.1% ATTO 550–DOPE was evaporated in a round‐bottomed flask, and the lipid film was dried under a vacuum overnight. The lipid film was hydrated with PBS at 20°C for 1 h. The lipid suspension was sonicated, subjected to four freeze/thaw cycles, and extruded 10 times through a 200 nm polycarbonate membrane (Avanti Research) to obtain small unilamellar vesicles. The phospholipid concentration of the small unilamellar vesicle solution was determined by the molybdenum blue method (Fiske and Subbarow 1925).

Then, 5 μm silica beads (Bangs Laboratories, Fishers, IN, USA) were cleaned using piranha solution (3:2 v/v H_2_SO_4_/H_2_O_2_) and thoroughly washed with PBS. The washed beads were mixed with the small unilamellar vesicle solution and incubated at 20°C for 1 h. The resulting beads were washed with PBS to remove excess vesicles.

### 4.6 CD47 Protein Purification and CD47/IgG‐Coated Bead Preparation

pFastBAC–CD47^ext^–His_10_ (Morrissey et al. 2020) was kindly provided by Dr. Morrissey, and CD47^ext^–His_10_ was produced by Sf9 cells and purified using Ni–NTA agarose beads (Fujifilm Wako). The supported lipid bilayer‐coated beads were suspended in PBS, and Alexa Fluor 647–anti‐biotin IgG and CD47^ext^–His_10_ were bound to achieve 300–3000 and 6000 molecules/μm^2^ density, respectively.

### 4.7 Phagocytosis Assay

Briefly, 6 × 10^4^ macrophage cells were seeded into a well of fibronectin‐coated Multiwell Glass‐Bottom Dish (D141400; Matsunami, Osaka, Japan), and 6 × 10^5^ of IgG‐ or IgG/CD47‐coated beads were added to each well after 4 h. To quantify the SIRPα–mEGFP localization, the cells were incubated with the beads for 15 min, fixed with 4% PFA, and stained with 2 μg/mL Hoechst 33342. Fluorescence images were acquired using a spinning disk confocal microscope [composed of IX83 (Evident, Nagano, Japan), CSU‐W1 (Yokogawa, Tokyo, Japan), LDI‐7 (89 North, Williston, VT, USA), and Zyla4.2 Plus (Andor, Belfast, United Kingdom)] and analyzed using ImageJ/Fiji. To capture the early stages of phagocytosis, phagocytic cups in which less than 50% of the bead surface was covered by the membrane were selected and analyzed. The phagocytic cup and the cell cortex were traced with a 10 pixel‐wide line at the Z‐slice with the clearest cross‐section of the cup. The average background intensity was measured in an adjacent region and subtracted from each measurement. More than 30 phagocytic cups from three independent experiments were manually quantified for all samples. Values are represented as dot plots and box plots created using R software, version 4.4.2 (The R Foundation for Statistical Computing).

To quantify phagocytosis, the cells were incubated with beads for 30 min, fixed with 4% PFA, and observed using an APX100 fluorescence microscope (Evident). For all samples, more than 300 cells from three independent experiments were manually counted.

When detecting phagocytosis by Raw 264.7 cells, we used Raw 264.7 cells infected with a lentiviral vector expressing EGFP.

### 4.8 Statistical Analysis

Comparisons between two groups were made using Student's *t*‐test except for Figure 4b. In Figure 4b, comparisons were made using the Mann–Whitney test. Differences among three groups were analyzed using one‐way ANOVA followed by Dunnett's test. Values of *p* less than 0.05 were considered statistically significant.

## Author Contributions

**Mihoko Kajita:** conceptualization, formal analysis, funding acquisition, investigation, methodology, validation, visualization, writing – original draft. **Yojiro Matsui:** formal analysis, investigation, methodology, validation, visualization. **Kotaro Sugimoto:** investigation, validation. **Shuto Takeuchi:** investigation, methodology. **Shota Matsumoto:** investigation, methodology. **Takahiro Okumura:** investigation, validation. **Hiroyuki Kajiura:** investigation. **Kazuki Motomura:** resources, visualization. **Atsushi Takeda:** resources, visualization. **Tomomi Koshiyama:** investigation, methodology, project administration, resources, writing – original draft. **Kyoko Shirakabe:** conceptualization, funding acquisition, project administration, resources, supervision, writing – original draft, writing – review and editing.

## Conflicts of Interest

The authors declare no conflicts of interest.

## Data Availability

The data that support the findings of this study are available from the corresponding author upon reasonable request.
